# *Enterococcus faecium* secreted the NlpC/P60 family protein to enhance host immunity and indirectly increases *Akkermansia muciniphila* for slowing aging

**DOI:** 10.3389/fmicb.2026.1680593

**Published:** 2026-01-29

**Authors:** Yinrui Guo, Shiqi Zou, Xieqing Yang, Ran Li, Weiqi Fu, Changqiong Xu, Fang Zhang, Xin Yang

**Affiliations:** 1 The Fifth Affiliated Hospital, School of Pharmaceutical Sciences, Guangzhou Medical University, Guangzhou, Guangdong, China; 2 Sun Yat-Sen Memorial Hospital, Sun Yat-Sen University, Guangzhou, Guangdong, China; 3 Clinical Medical College, Yichun University, Yichun, Jiangxi, China; 4 Hunan Yueyang Maternal & Child Health-Care Hospital, Yueyang, Hunan, China

**Keywords:** *Akkermansia muciniphila*, anti-aging, *Enterococcus faecium*, host immunity, NlpC/P60 family protein

## Abstract

While probiotics like *Enterococcus faecium* are known for gut health benefits, their potential anti-aging effects are poorly understood. This study investigated whether *E. faecium* fermentation broth delays aging and explored its mechanisms. Using a mouse model, lifespan assays suggested that the *E. faecium fermentation* broth may contribute to lifespan extension, indicating anti-aging properties. Microbiome analysis showed it modulated gut microbiota, increasing beneficial *Akkermansia* abundance. Key active components identified included myo-inositol (promoting hair follicle growth), D-ribose, and secreted proteins. While myo-inositol increased the abundance of *Lactobacillus reuteri* and *Lactobacillus johnsonii*, it did not increase *Akkermansia*. A high-content secreted protein, NlpC/P60, present in *E. faecium* fermentation broth, may enhance host immunity through the NOD-like receptor signaling pathway, thereby restricting pathogen colonization and reshaping the gut microbiota. This immune boost indirectly elevated levels of beneficial bacteria like *Akkermansia muciniphila* and *L. johnsonii*, improving overall microbiota composition and mitigating age-related diseases. The findings demonstrate that *E. faecium* fermentation broth combats aging through multiple pathways, primarily microbiota modulation and immune enhancement. The identification of NlpC/P60 as a key mediator provides crucial mechanistic insight. This study elucidates the material basis and pathways by which *E. faecium* fermentation broth delays aging, offering experimental support for developing novel microecological therapies against age-related diseases.

## Introduction

Aging is accompanied by alterations in gut microbiota, which in turn influence the rate of aging (Boehme et al., 2021; Ghosh et al., 2022; Parker et al., 2022; Lu et al., 2025). Probiotics and their metabolites have emerged as potential intervention strategies to delay aging (Plovier et al., 2017; Hou et al., 2023; Zhou et al., 2025). A positive correlation exists between “longevity-adapted” and health-associated gut bacteria (e.g., *Akkermansia, Bifidobacterium*, and *Christensenellaceae*) (Biagi et al., 2016). Compared to younger adult groups, centenarians exhibit enrichment of bacteria such as the genera *Akkermansia, Parabacteroides, Alistipes*, and *Odoribacter*, which are associated with anti-inflammatory activity, weight loss, and reduced metabolic disorders (Pang et al., 2023). Supplementation with probiotics has been utilized to modulate gut microbiota and physiological functions to delay host aging. Studies have shown that the genus *Bifidobacterium* is strongly negatively correlated with host age, and *Bifidobacterium longum* was found to delay aging in mice through the bacterial arginine biosynthesis pathway (Xiao et al., 2021). Supplementation with *A. muciniphila* promotes healthy aging and extends lifespan in aged mice (Barcena et al., 2019; Cerro et al., 2022). The probiotic *Lactobacillus rhamnosus* Probio-M9 shows potential as a dietary supplement to delay aging in *Caenorhabditis elegans* (Zhang et al., 2022). Supplementation with the single beneficial probiotic Bifidobacterium longum improves fracture healing in aged (18-month-old) female mice (Roberts et al., 2023). Professor Anchalee Prasansuklab's team demonstrated that the probiotic *Lactobacillus parac*asei HII01, as a dietary supplement, maintains health status and extends lifespan (Kumaree et al., 2023). These studies collectively suggest that probiotics, as microecological agents, possess potential functions in delaying aging and prolonging lifespan. However, the specific mechanisms of action and material basis remain largely unelucidated.

*Enterococcus faecium* is a bacterial species with a dual nature: while certain strains [e.g., *NCIMB 10415* (Palkovicsné Pézsa et al., 2022)*, also known as SF68* (Franz et al., 2024)] have been used as probiotics in veterinary and human applications, the species is more widely recognized in clinical settings as a leading cause of nosocomial infections, particularly among immunocompromised or critically ill patients (Arias and Murray, 2012; Wei et al., 2024). *E. faecium* isolated from chicken cecum exhibits immunomodulatory and longevity-promoting effects in *C. elegans* (Sim et al., 2018). Specific strains of *E. faecium* could cause Enterococcal bacteremia (Sörstedt et al., 2024). Different strains of *E. faecium* could protect *C. elegans* against enteric pathogens, and secreted antigen A (SagA) from *E. faecium* could also be heterologously expressed and secreted to improve the protective activity of probiotics against Salmonella pathogenesis in *C. elegans* and mice (Rangan et al., 2016). A unique peptidoglycan hydrolase, secreted antigen A (SagA) from *E. faecium*, can enhance intestinal barrier function and confer tolerance against *Salmonella enterica serotype Typhimurium* and *Clostridium difficile* pathogenesis in multiple mouse models (Pedicord et al., 2016). Certain Enterococci, especially *E. faecium*, express and secrete orthologs of the NlpC/p60 peptidoglycan hydrolase SagA, which is crucial for enhancing the effectiveness of immune therapy in the mouse models (Griffin et al., 2021). Therefore, different strains of *E. faecium* may have significantly different effects.

While numerous gut microbes have been implicated in modulating host aging, we focused on *E. faecium* due to emerging evidence that certain strains of this species can promote intestinal homeostasis, enhance immune function, and extend lifespan in model organisms—despite its well-documented role as an opportunistic pathogen in clinical settings. Given this context, safety considerations are paramount when exploring E. faecium-derived interventions. In this study, we specifically utilize cell-free fermentation broth—and, in follow-up experiments, purified secreted proteins—rather than live bacteria, to harness potential health benefits while mitigating risks associated with live microbial administration. This study aims to evaluate the anti-aging effects of *E. faecium* and its fermentation broth in aged mouse models, and to further elucidate the specific active components responsible for delaying aging as well as their underlying mechanisms of action.

## Methods

### Preparation of living bacteria and fermentation broth

Under aerobic conditions, a monoclonal colony of *E. faecium* was inoculated into 10 mL of MRS medium and cultured overnight at 37°C. A 100 μL aliquot of the bacterial culture was transferred to a 500 mL Erlenmeyer flask containing 200 mL of MRS broth, followed by incubation at 37°C with continuous shaking (200 rpm) until the OD_600_ reached approximately 1. The *E. faecium* culture used for the live bacteria group was standardized to an optical density of OD600 = 1.0, which corresponded to approximately 1.0 × 10^9^ CFU/mL, as determined by serial dilution and plating on MRS agar followed by anaerobic incubation at 37°C for 48 h.

Live bacterial suspension: The culture broth was centrifuged at 7,000 rpm (RCF = 5,150 × g) for 10 min to collect the bacterial pellet, which was washed twice with PBS and resuspended in PBS to adjust the OD_600_ to approximately 1. Subcultured weekly to maintain bacterial viability. The supernatant was centrifuged (10,000 × g, 15 min) to remove bacterial cells, and the supernatant was filtered through a 0.22 μm polyethersulfone membrane filter (Millipore). Sterility was verified by inoculating 100 μL of the filtered broth onto MRS agar plates and incubating anaerobically at 37°C for 72 h; absence of colony growth confirmed successful removal of viable bacteria. Stored at 4°C for future use.

### Mice

All the experimental protocols were approved by the Guangzhou BGsciences Biotechnology Co., Ltd. and the mice were obtained from the Guangzhou Ruige Biological Technology (Production License: SCXK [Yue] 2023-0059; Institutional Animal Use License: SYXK [Yue] 2023-0343). The detailed scheme of the experimental design is described in the figure. All mice were housed in a specific pathogen-free animal facility, maintained on a 12 h light/12 h dark cycle, at a temperature of 22°C and 45% humidity, with *ad libitum* access to food and water. Description of several animal experiments.

Experiment 1: SPF-grade male C57BL/6 mice (aged 710 days) were used. Experimental groups were divided as follows: (1) Control group (*n* = 4): administered 0.15 mL/mouse of PBS via oral gavage every other day; (2) Live bacteria group (*n* = 5): treated with 0.15 mL/mouse of *E. faecium* live bacterial suspension (OD_600_ = 1) on the same schedule; (3) Fermentation broth group (*n* = 5): administered 0.15 mL/mouse of bacterial fermentation broth every other day.

Experiment 2: SPF-grade female C57BL/6 mice (17-month-old) were used in this study. The animals were divided into two groups: (1) Control group (*n* = 10), receiving 0.15 mL/mouse of PBS via oral gavage every other day; (2) Fermentation broth group (*n* = 10), administered 0.15 mL/mouse of bacterial fermentation broth following the same dosing schedule. Both groups were maintained under standardized conditions to ensure experimental consistency.

Experiment 3 [Constipation model in aged mice (Wei et al., 2023)]: 10-month-old SPF-grade male KM mice were used in this study. The animals were divided into experimental groups as follows: (1) Normal group (*n* = 6): Received 0.2 mL/mouse of PBS via oral gavage without any additional treatment. (2) The remaining mice first underwent a 1-week modeling period with loperamide (5 mg/kg/day, Xian Janssen Pharmaceutical Ltd.) to establish the disease model, then were divided into three groups (*n* = 6 each): Control group: Administered 0.2 mL/mouse of PBS daily by gavage; Positive control group: Treated with 0.2 mL/mouse of lactulose oral solution (1 mL:557 mg, Hanmi Pharm. Co., Ltd.); Fermentation broth group: Received 0.15 mL/mouse of fermentation broth daily. All groups continued their respective treatments for an additional week following the modeling period.

Experiment 4: SPF-grade KM mice (10-month-old) were used. Hair removal was performed with 5% sodium sulfide. Animal grouping and drug administration were as follows: (1) Control group (*n* = 6): Administered PBS via oral gavage (0.2 mL/mouse) every other day. (2) Inositol group (*n* = 6): Administered protein solution (150 mg/mL, 0.2 mL/mouse) via oral gavage every other day.

Experiment 5: SPF-grade C57BL/6 mice aged 18 months were used. Animal grouping and administration protocols: Control group (*n* = 6) received PBS via oral gavage (0.2 mL/mouse) every other day; Protein group (*n* = 6) received protein solution (0.2 mL/mouse at 50 μg/mL concentration) via oral gavage every other day. For details on how the protein was prepared, please refer to the Supplementary Data.

Anesthesia was induced with sodium pentobarbital (3 mg/mL) administered intraperitoneally (50 mg/kg). At the end of the experiment, animals were euthanized by cervical dislocation.

These experiments form a logical cascade: from initial safety and efficacy screening (Exp. 1–2), to disease-relevant functional validation (Exp. 3), and finally to component identification and mechanistic refinement (Exp. 4–5).

### Histopathology and immunostaining

The tissue specimens were immediately fixed in 4% paraformaldehyde (pH 7.4) for 24 h to preserve morphological integrity. Following fixation, samples underwent sequential processing: dehydration through a graded ethanol series (70–100%), clearing in xylene, and paraffin infiltration at 60°C. Embedded tissues were sectioned at 3 μm thickness using a rotary microtome (Leica RM2235).

For hematoxylin and eosin (H&E) staining:

Sections were deparaffinized in xylene (2 × 5 min), rehydrated through a descending ethanol series (100% → 70%), and rinsed in distilled water. Nuclei were stained with Mayer's hematoxylin for 3–5 min, differentiated in acid alcohol, blued in ammonia water, and counterstained with eosin Y for 1–2 min. Slides were dehydrated, cleared in xylene, and mounted with neutral balsam.

For immunohistochemical (IHC) staining, the two-step polymer-based detection system (Dako, K8002) was employed:

Deparaffinization and rehydration as above. Antigen retrieval: Slides were subjected to heat-induced epitope retrieval (HIER) in citrate buffer (10 mM sodium citrate, pH 6.0) or EDTA/Tris buffer (pH 9.0), depending on the target antigen, using a pressure cooker, microwave, or water bath at 95–100°C for 20–30 min, followed by gradual cooling to room temperature. Endogenous peroxidase blocking: Incubation with 3% (v/v) hydrogen peroxide (H_2_O_2_) in PBS for 10–15 min at room temperature to quench endogenous peroxidase activity. Blocking: Non-specific binding sites were blocked with 5–10% normal serum (from the species of the secondary antibody) or BSA for 30 min. Primary antibody incubation: Slides were incubated with validated primary antibodies overnight at 4°C in a humidified chamber. Secondary antibody and detection: After washing in PBS (3 × 5 min), slides were incubated with HRP-conjugated polymer secondary antibody for 30–60 min at room temperature. The slides were washed 2–3 times in PBS for 5 min each on a decolorizing shaker. Chromogen development: Antigen-antibody complexes were visualized using 3,3′-diaminobenzidine (DAB) substrate, producing a brown precipitate at the site of target expression. Reaction was monitored under a microscope and stopped with distilled water. Counterstaining and mounting: Nuclei were lightly counterstained with Mayer's hematoxylin (30–60 s), blued, dehydrated through graded alcohols, cleared in xylene, and coverslipped with neutral balsam.

### RNA isolation and sequencing

RNA concentration and purity was measured using NanoDrop 2000 (Thermo Fisher Scientific, Wilmington, DE). RNA integrity was assessed using the RNA Nano 6000 Assay Kit of the Agilent Bioanalyzer 2100 system (Agilent Technologies, CA, USA). A total amount of 1 μg RNA per sample was used as input material for the RNA sample preparations. Sequencing libraries were generated using the NEBNext UltraTM RNA Library Prep Kit for Illumina (NEB, USA) following manufacturer's recommendations and index codes were added to attribute sequences to each sample. Briefly, mRNA was purified from total RNA using poly-T oligo-attached magnetic beads. Fragmentation was carried out using divalent cations under elevated temperature in NEBNext First Strand Synthesis Reaction Buffer (5X). First-strand cDNA was synthesized using random hexamer primer and M-MuLV Reverse Transcriptase. Second-strand cDNA synthesis was subsequently performed using DNA Polymerase I and RNase H. Remaining overhangs were converted into blunt ends via exonuclease/polymerase activities. After adenylation of 3′ ends of DNA fragments, NEBNext Adaptor with hairpin loop structure was ligated to prepare for hybridization. In order to select cDNA fragments of preferentially 240 bp in length, the library fragments were purified with AMPure XP system (Beckman Coulter, Beverly, USA). Then 3 μL USER Enzyme (NEB, USA) was used with size-selected, adaptor-ligated cDNA at 37°C for 15 min followed by 5 min at 95°C before PCR. Then PCR was performed with Phusion High-Fidelity DNA polymerase, Universal PCR primers and Index (X) Primer. At last, PCR products were purified (AMPure XP system) and library quality was assessed on the Agilent Bioanalyzer 2100 system. The clustering of the index-coded samples was performed on a cBot Cluster Generation System using TruSeq PE Cluster Kit v4-cBot-HS (Illumina) according to the manufacturer's instructions. After cluster generation, the library preparations were sequenced on an Illumina platform and paired-end reads were generated.

### Western blotting

At the endpoint of each experiment, mice were euthanized under deep anesthesia, and target tissues (e.g., colon, liver, skin) were rapidly dissected. Tissues were immediately snap-frozen in liquid nitrogen and stored at −80°C until use. Tissues were homogenized using a motorized tissue homogenizer in ice-cold RIPA lysis buffer (Thermo Scientific™, Cat. No. 78510) supplemented with protease and phosphatase inhibitor cocktails (Solarbio, P1261). The homogenates were then incubated on ice for 30 min to ensure complete lysis, with brief vortexing every 10 min, followed by centrifugation at 12,000 × g for 15 min at 4°C. The resulting supernatants were carefully collected, and total protein concentration was determined using a BCA Protein Assay Kit (Solarbio, PC0020) according to the manufacturer's instructions, with bovine serum albumin (BSA) as the standard. Equal amounts of protein (typically 35 μg per lane) were mixed with 4 × Laemmli sample buffer (containing β-mercaptoethanol), denatured at 95°C for 5 min, and loaded onto 8–12% SDS-polyacrylamide gels. Electrophoresis was performed at a constant voltage (80–120 V) until dye front migration was complete. Proteins were then electrophoretically transferred onto 0.45 μm PVDF membranes using a wet transfer system at 100 V for 60–90 min, with cooling to prevent overheating. After blocking with 5% non-fat dry milk in TBST (20 mM Tris-HCl, 500 mM NaCl, 0.2% Tween-20, pH 7.4), membranes were incubated with primary antibodies overnight at 4°C. The specific primary antibodies, catalog numbers, and working concentrations are listed in Supplementary Information. After three 10-min washes with TBST, membranes were incubated with HRP-conjugated secondary antibodies (Servicebio, 1:3,000) for 1 h at room temperature. Following additional TBST washes, immunoreactive bands were visualized using enhanced chemiluminescence (ECL) substrate and detected with a chemiluminescent imaging system (Tanon). Band intensities were quantified using ImageJ software (NIH, USA), normalized to loading controls (e.g., β-actin or GAPDH), and expressed relative to the control group.

### Microbiome analysis

Total DNA was extracted from 250–500 mg of colonic content samples using the QIAamp DNA Stool Mini Kit (Qiagen, Germany), and its purity and concentration were measured with a NanoDrop 2000 spectrophotometer (Thermo Fisher Scientific, USA). The microbial 16S rRNA genes were amplified using primers (forward: 5′-ACTCCTACGGGAGGCAGCA3′; reverse: 5′-GGACTACHVGGGTWTCTAAT3′) designed from conserved regions, followed by PCR amplification, purification, and library construction. Qualified libraries were sequenced on the Illumina HiSeq 2500 platform, and raw sequencing data were processed through Base Calling, with results stored in FASTQ format containing sequence reads and quality information.

### LC-MS untargeted metabolomics

Untargeted metabolomics is a frequently used research method in metabolomics studies. Its primary research approach involves comparing experimental and control groups, detecting metabolites present in the samples, and obtaining quantitative information to identify statistically significant differential metabolites between different groups. These findings can help explain the associations between the identified metabolites and biological processes or states. The chromatographic column used is the Waters ACQUITY Premier HSS T3 Column with a particle size of 1.8 μm and dimensions of 2.1 mm ^*^ 100 mm. Mobile phase A consists of 0.1% formic acid in water, while mobile phase B is 0.1% formic acid in acetonitrile. The column temperature is set at 40°C, with a flow rate of 0.4 mL/min and an injection volume of 4 μL. Mass spectrometry detection is performed using the AB TripleTOF 6600. The raw mass spectrometry data are converted to mzXML format using ProteoWizard, and peak extraction, alignment, and retention time correction are carried out using the XCMS program. Peaks with a missing rate >50% in each group of samples are filtered out, and KNN imputation is performed for missing values. The peak areas are corrected using the SVR method. The corrected and filtered peaks are then identified as metabolites by searching a self-built laboratory database, integrating public and predictive databases, and utilizing the metDNA approach. Finally, substances with a comprehensive identification score above 0.5 and a CV value less than 0.3 in QC samples are extracted. These substances are then merged from positive and negative modes (retaining substances with the highest qualitative grade and the smallest CV value) to obtain the ALL_sample_data file, which can be found in the Supplementary Table.

### Label-free quantitative proteomics

Label-free is a non-labeling quantitative proteomics technique that does not require the use of isotope labels as internal standards. Instead, it directly performs relative quantification of proteins based on the intensity of peptide mass spectrometry peaks. Proteins extracted from different samples are quantified, and equal amounts of protein from each sample are subjected to trypsin digestion. The purified peptide fractions from each group are then analyzed separately by LC-MS/MS. Specific software is used to search the raw mass spectrometry data to extract quantitative information for each peptide in different samples, thereby obtaining qualitative and quantitative information for proteins across the different samples. The Label-free quantitative proteomics data for *E. faecium* fermentation broth can be found in the Supplementary Table.

### Measurement of bacterial growth rate

*Akkermansia muciniphila* (ATCC BAA-835) was used. Under anaerobic conditions (85% N_2_, 10% H_2_, and 5% CO_2_) in a workstation, purified protein (0.4 mg/mL, variable volumes) and 10 μL *A. muciniphila* inoculum (OD600 = 0.155) were mixed, then brought to a total volume of 1,800 μL with BHI medium with 5% FBS. The experiment included three biological replicates. After 24 h, 120 μL aliquots were transferred from each tube to a 96-well plate for OD_600_ measurement.

### Statistical analysis

Data are described as means ± the standard deviation of at least three independent experiments. Datasets that involved more than two groups were analyzed via one-way analysis of variance using Statistical Package for the Social Sciences software (SPSS version 17.0, Abacus Concepts, Berkeley, CA, USA) or Prism8 software (GraphPad, San Diego, CA, USA). *Post-hoc* comparisons were performed using Fisher's Least Significant Difference test.

## Results

### *E. faecium* fermentation broth extends lifespan and ameliorates multi-organ aging and gut microbiota in aged mice

**Figure 1:**
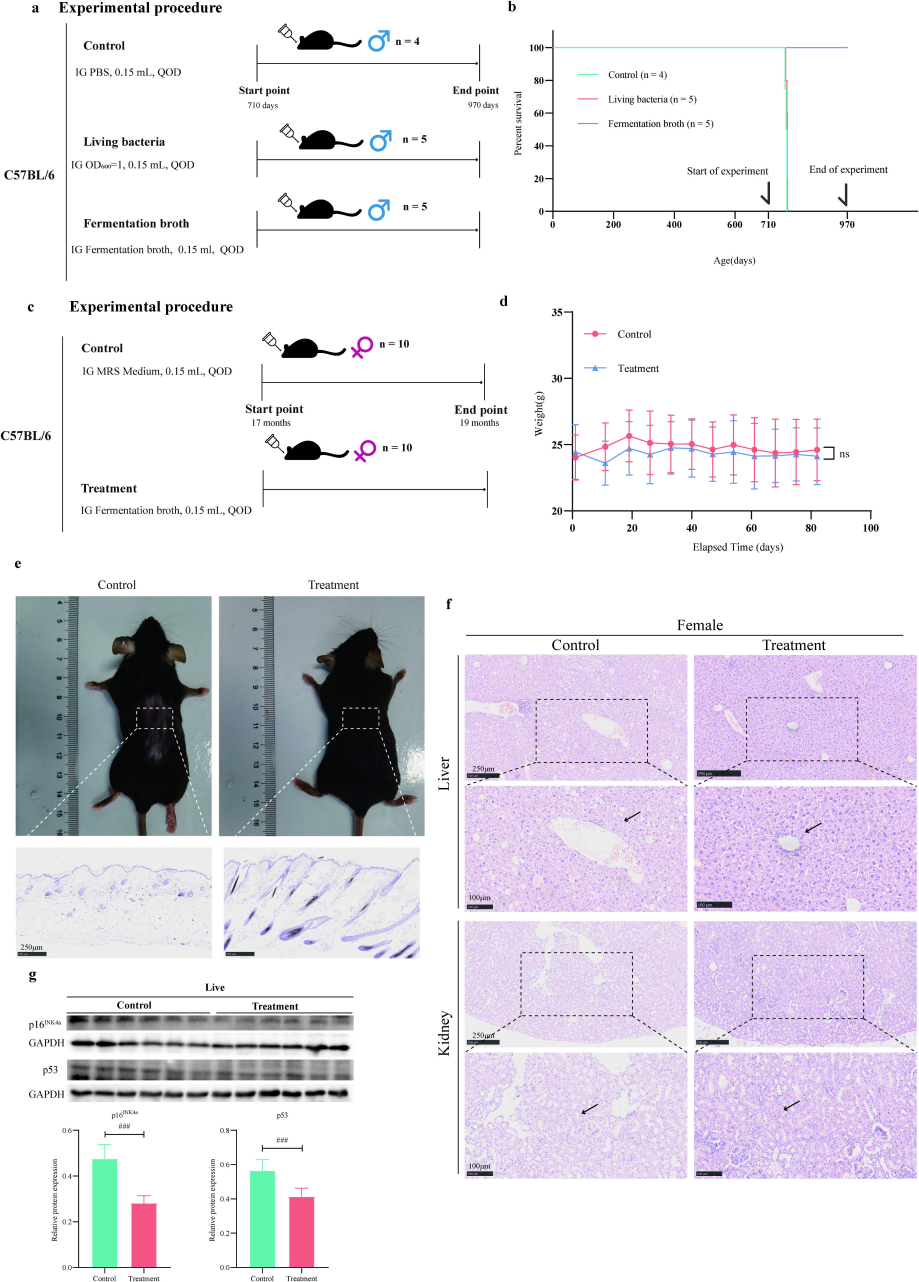
*E. faecium* fermentation broth can effectively delay aging in multiple organs. **(a)** Schematic of the animal experimental design and group allocation. **(b)** The survival curve of aged mice. **(c)** Schematic of the animal experimental design and group allocation. **(d)** Changes in body weight of aged mice. **(e)** Hair growth status in aged mice and H&E staining results of dorsal skin. **(f)** HE staining images of liver and kidney tissue sections from aged mice. **(g)** The expression levels of senescence markers p16^INK4A^ and p53 in mouse liver tissues were detected by Western blotting. Data are resented as mean ± SD from six independent experiments. ^#^*p* < 0.05, ^##^*p* < 0.01, ^###^*p* < 0.001, determined by two-tailed Student's *t*-test.

**Figure 2:**
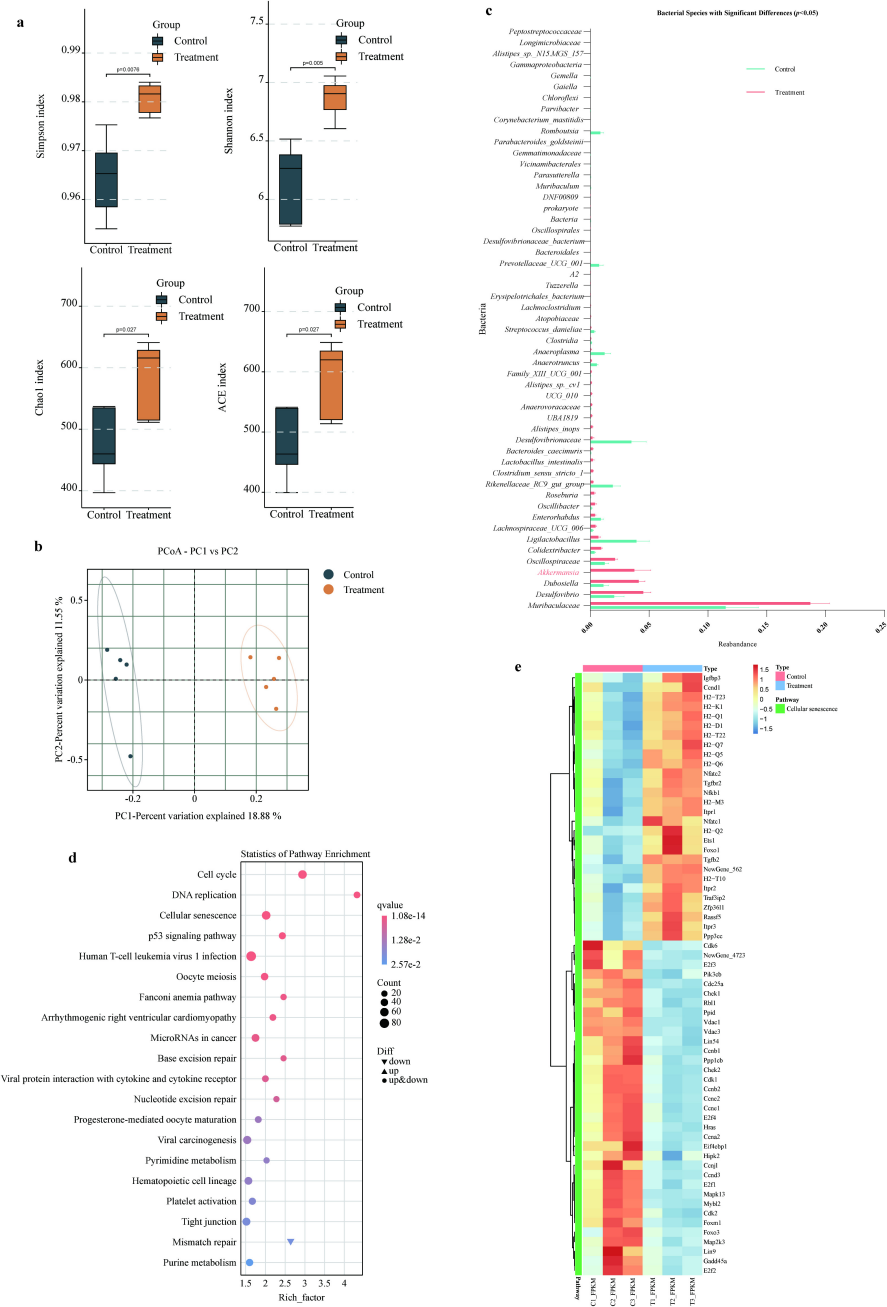
*E. faecium* fermentation broth modulates gut microbiota and delays splenic tissue aging. **(a)** Comparative analysis of alpha diversity indices (Each group: *n* = 5, statistical significance assessed using Student's *t*-test). **(b)** The principal coordinate analysis (PCoA) was conducted using the binary jaccard distance metric. In the resulting plot, each data point corresponds to an individual sample, with distinct colors representing different experimental groups. The elliptical boundaries depict 95% confidence intervals, indicating that 95 out of 100 hypothetical samples from the same group would be expected to fall within these regions. The x-axis displays the first principal coordinate along with its percentage contribution to the observed variance, while the y-axis similarly represents the second principal coordinate and its proportional explanation of sample variation. **(c)** Metastats analysis was employed. The figure displays the results with significant differences at the species level between the groups (*p* < 0.05). **(d)** Enrichment analysis of splenic differentially expressed genes in KEGG pathway between the control group and the treatment group. **(e)** The heatmap of differentially expressed genes in the cellular senescence signaling pathway in splenic tissues between the control and treatment groups.

The research group previously isolated bacterial strains from human fecal samples and identified a strain capable of extending the lifespan of *C. elegans*, which was characterized as *E. faecium* (Supplementary Figure S1). Existing literature has demonstrated that *E. faecium* can extend the lifespan of *C. elegans* (Sim et al., 2018). The lifespan-extending effects of *E. faecium* observed in *C. elegans* suggest its potential as a probiotic strain for modulating host longevity. As *E. faecium* is a common gut commensal bacterium, its longevity-promoting mechanisms may involve: (1) production of bioactive metabolites, (2) suppression of pathogenic bacteria, or (3) activation of host stress-response pathways. Further studies should validate these mechanisms in mammalian models and evaluate their safety for potential human applications. To further elucidate the anti-aging effects of *E. faecium* and its fermentation products, we conducted intervention experiments using aged mice (710 days old) with live bacteria and fermented broth (Figure 1a). The results demonstrated that the *E. faecium fermentation* broth may contribute to lifespan extension of the mice compared with the control group (Log-rank (Mantel-Cox) test, *P* = 0.0040) and the live bacteria group did not extend the lifespan of aged mice compared with the control group [Log-rank (Mantel-Cox) test, *P* = 0.0988; Figure 1b]. Therefore, we further conducted pharmacodynamic experiments to evaluate the anti-aging effects of *E. faecium* fermentation broth (Figure 1c). The experimental results showed that, compared with the control group, the fermentation broth group did not alter the body weight of the mice (Figure 1d). Experimental results demonstrated that, compared with the control group, the fermentation broth group significantly ameliorated age-related alopecia and kept fur darkening (Figure 1e and Supplementary Figure S2a). Histopathological examination of renal and hepatic tissues by HE staining is presented in Figure 1f. Renal HE staining results revealed that control group specimens exhibited predominantly dilated tubular lumens with increased intertubular spacing, while the fermentation broth group demonstrated narrow, regularly-shaped tubular lumens within the visual field, suggesting potential anti-aging effects on renal tissue. Hepatic HE staining results showed that control group displayed disorganized hepatocyte arrangement with irregular morphology and indistinct boundaries, accompanied by extensive fatty degeneration, pale and loose cytoplasm containing round vacuoles, and partial nuclear pyknosis. In contrast, the fermentation broth group maintained preserved hepatocyte morphology with orderly arrangement near central veins, although cellular distribution appeared disorganized in peripheral regions, along with relatively distinct hepatic cord structures. The Western blot analysis results of hepatic senescence markers p53 and p16^INK4A^ are shown in Figure 1g. The results demonstrate that the fermentation broth group significantly reduced the expression levels of both p53 and p16^INK4A^ in liver tissues, indicating that the *E. faecium* fermentation broth effectively delays hepatic senescence. The alpha diversity indices analysis revealed significant differences in Simpson, Shannon, Chao1, and ACE (Figure 2a), demonstrating that the fermentation broth could modulate the composition of gut microbiota. The PCoA analysis of β-diversity revealed distinct clustering patterns between the control and fermentation broth groups (Figure 2b), indicating that the fermentation broth possesses gut microbiota-modulating properties. Metastats analysis of intergroup microbial composition differences demonstrated that the fermentation broth group significantly increased the abundance of *Akkermansia* (Figure 2c). KEGG pathway enrichment analysis of splenic transcriptomes between the two groups revealed significant associations with the following signaling pathways: Cell cycle, DNA replication, Cellular senescence, and p53 signaling pathway (Figure 2d and Supplementary Figure S2b). The heatmap of differentially expressed genes in the cellular senescence signaling pathway between control and treatment groups in splenic tissues (Figure 2e) demonstrated that the fermentation broth exerts anti-aging effects.

### *E. faecium* fermentation broth alleviates constipation and modulates gut microbiota in aged constipated mice

**Figure 3:**
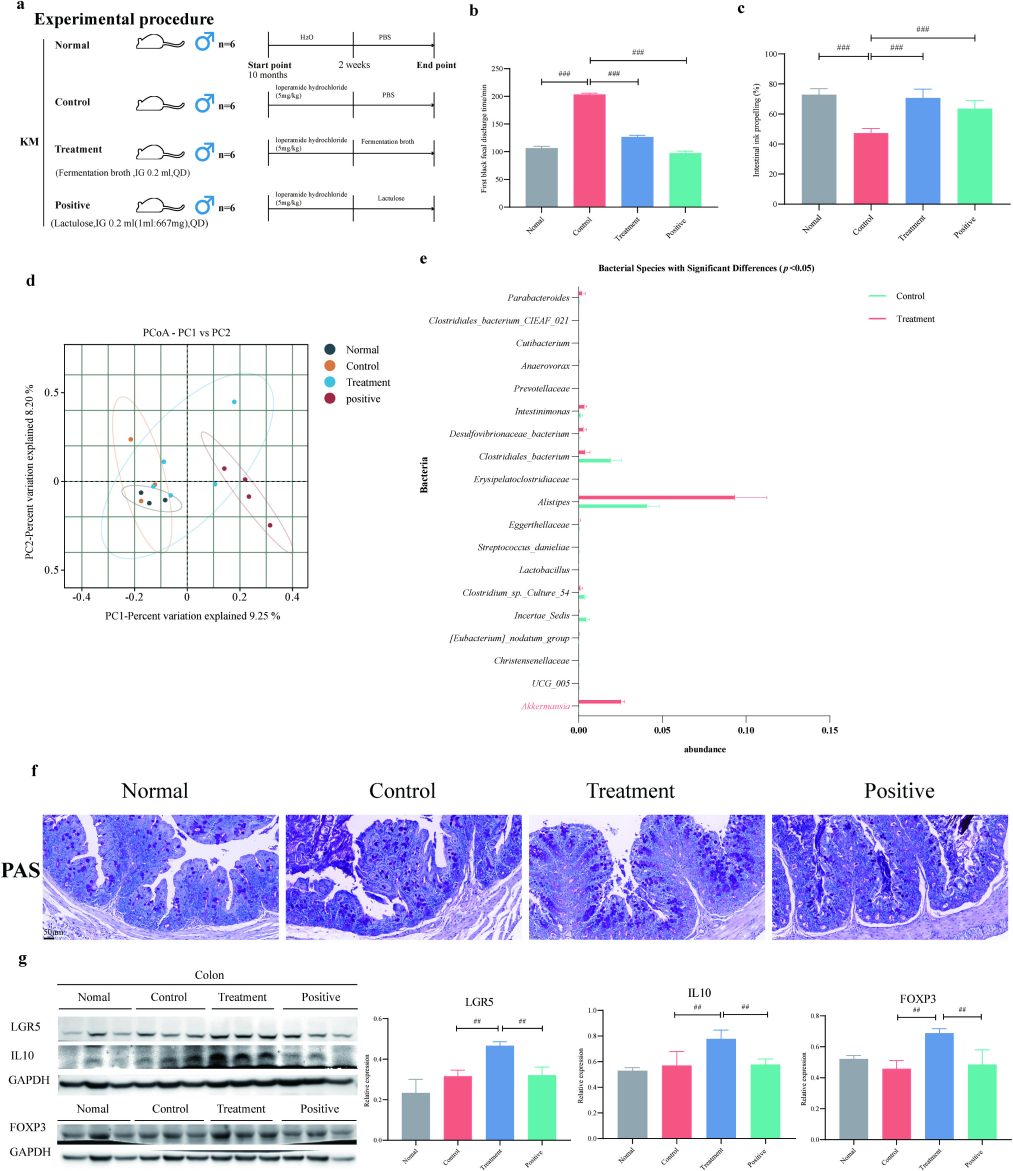
*E. faecium* fermentation broth alleviates age-related constipation and modulates gut microbiota. **(a)** Schematic of the animal experimental design and group allocation. **(b)** The time to first black stool excretion in aged constipated mice. Data are presented as mean ± SD from six independent experiments. ^#^*p* < 0.05, ^##^*p* < 0.01, ^###^*p* < 0.001, determined by one-way analysis of variance. **(c)** The intestinal ink propulsion rate in aged constipated mice. **(d)** The principal coordinate analysis (PCoA) was conducted using the binary jaccard distance metric. **(e)** Metastats analysis was employed. The figure displays the results with significant differences at the species level between the groups (*p* < 0.05). **(f)** PAS staining results of colon tissue sections. **(g)** The expression levels of LGR5, IL10, and FOXP3 in mouse colon tissues were detected by Western blotting. Data are presented as mean ± SD from three independent experiments. ^#^*p* < 0.05, ^##^*p* < 0.01, ^###^*p* < 0.001, determined by one-way analysis of variance.

We further established a constipation model in aged KM mice using loperamide hydrochloride to evaluate whether the *E. faecium* fermentation broth could ameliorate senile constipation and regulate the intestinal microbiota (Figure 3a). The results are shown in Figures 3b, c. Both the fermentation broth group and the positive drug group significantly shortened the time to the first black stool defecation (*p* < 0.05) and significantly increased the small intestinal ink propulsion rate (*p* < 0.05) in mice. Therefore, the fermentation broth group exhibited an ameliorative effect on senile constipation. The PCoA results of β-diversity are shown in Figure 3d. The results indicate that there are differences in microbial community structure among the groups, suggesting that the fermentation broth has a certain regulatory effect on the gut microbiota. Metastats analysis was performed to compare microbial differences between the control group and the fermentation broth group. The results showed that the fermentation broth significantly increased the abundance of *Akkermansia* (Figure 3e). *E. faecium* can ameliorate intestinal barrier damage (Wu et al., 2019). Its secreted NlpC/p60 family protein plays a crucial immunomodulatory role by activating innate immunity, inducing an immune-favorable microenvironment, and enhancing the efficacy of immunotherapy (Griffin et al., 2021). Additionally, it strengthens host immunity and limits bacterial pathogenesis (Rangan et al., 2016; Kim et al., 2019). The PAS staining results of the colon tissue are shown in Figure 3f. The results indicate that the number of goblet cells in the control group was significantly lower than that in the fermentation broth group, suggesting that the fermentation broth may have the effect of increasing goblet cells in the colon tissue. KEGG enrichment analysis of the colonic transcriptome revealed an association with the Fc gamma R-mediated phagocytosis signaling pathway, suggesting that the pharmacological effects of *E. faecium* fermentation broth may involve immunomodulation (Supplementary Figure S2c). Immune cells and their secreted factors can regulate the proliferation and differentiation of intestinal stem cells (ISCs; Biton et al., 2018; Hou et al., 2020). The expansion and differentiation of ISCs replace damaged intestinal epithelial cells, thereby improving gut function (Hou et al., 2020). Western blot analysis of colonic tissues revealed the expression levels of the intestinal stem cell marker LGR5, as well as related immune cells and their associated factors. The results demonstrated that the fermentation broth group significantly upregulated the expression of LGR5, IL10, and FOXP3 in colonic tissues (Figure 3g). These findings suggest that the fermentation broth of *E. faecium* can increase the number of goblet cells and intestinal stem cells, improve the gut microbiota, and alleviate age-related functional constipation.

### A protein secreted by *E. faecium* increases the abundance of *Akkermansia*

To further identify the anti-aging active components in *E. faecium* fermentation broth, LC-MS/MS coupled with label-free quantitative proteomic analysis was performed for substance identification, with the results presented in Supplementary Figures S3, S4. The inositol and D-ribose present in the fermentation broth may have potential benefits in delaying aging. Scientific studies support inositol's role in anti-aging (Shi et al., 2023), improving metabolic syndrome and diabetes (Celentano et al., 2016), aiding in the treatment of polycystic ovary syndrome (PCOS; Facchinetti et al., 2020), and enhancing hair quality (Zhang et al., 2024). D-Ribose has been shown to benefit heart health (Pauly and Pepine, 2000; Omran et al., 2003), exercise recovery, and fatigue resistance (Teitelbaum et al., 2006; Seifert et al., 2017). Therefore, we further evaluated the effects of inositol, a highly abundant product in the fermentation broth, on promoting hair growth and modulating gut microbiota. The experimental design was illustrated in Supplementary Figure S5a. The results demonstrated that inositol supplementation promoted hair growth in aged KM mice (Supplementary Figure S5b). The PCoA analysis of β-diversity revealed distinct clustering patterns between the control and inositol groups (Supplementary Figure S5c), indicating that the inositol could regulate gut microbiota. Metastats analysis further compared species-level differences between the two groups. While inositol did not significantly increase the abundance of *Akkermansia*, it enhanced the abundance of *L. reuteri* and *L. johnsonii* which were commonly regarded as probiotics (Supplementary Figure S5d).

**Figure 4:**
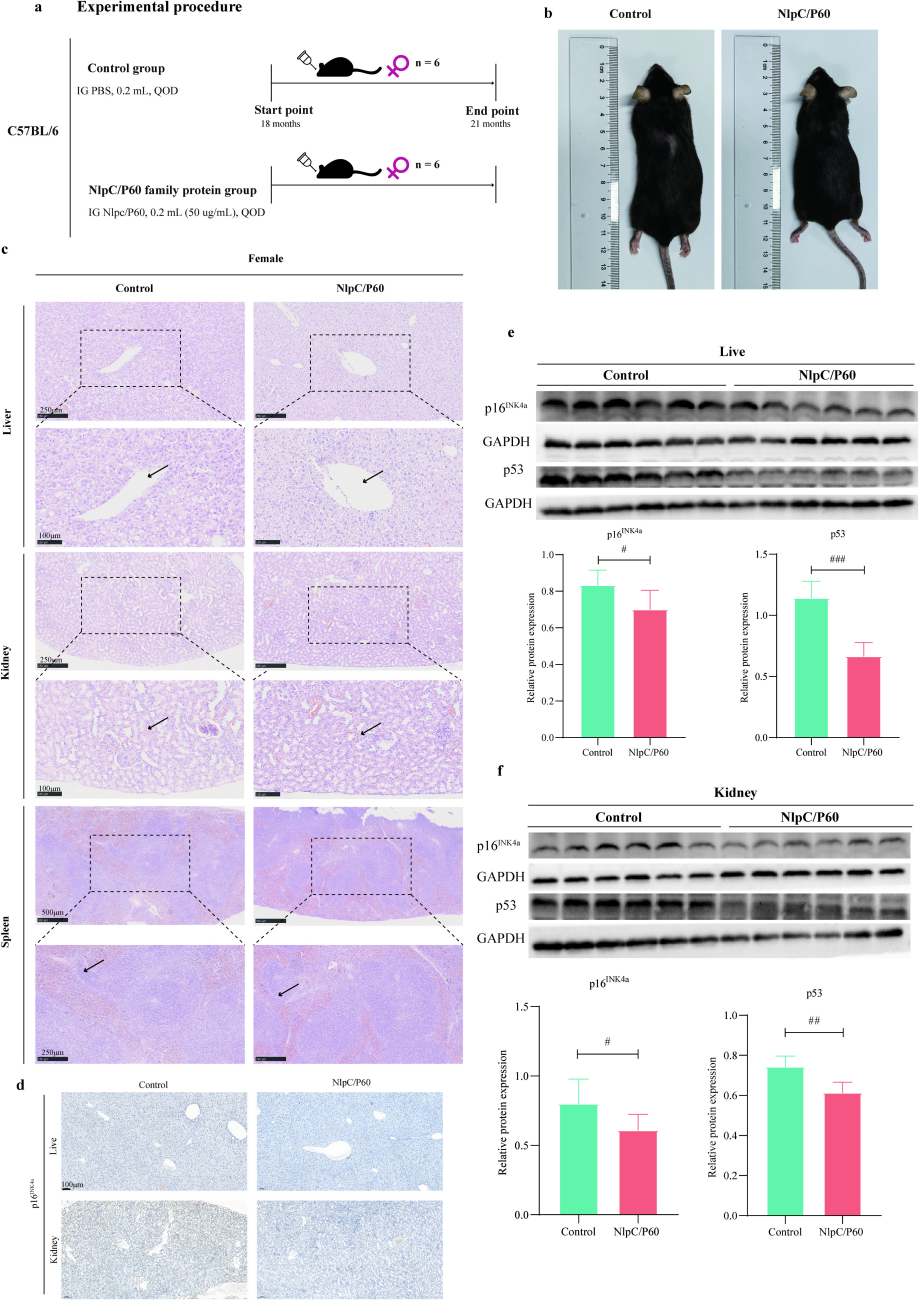
The NlpC/P60 family protein derived from *E. faecium* fermentation broth mitigates multi-organ aging in mice. **(a)** Schematic of the animal experimental design and group allocation. **(b)** Hair growth status in aged mice. **(c)** HE staining images of liver, kidney and spleen tissue sections from aged mice. **(d)** The expression of p16^INK4A^ in liver and kidney tissue were detected by immunohistochemistry. **(e,f)** The expression of senescence markers p16^INK4A^ and p53 in liver and kidney tissues was detected by Western blot. Data are presented as mean ± SD from six independent experiments. ^#^*p* < 0.05, ^##^*p* < 0.01, ^###^*p* < 0.001, determined by two-tailed Student's *t*-test.

**Figure 5:**
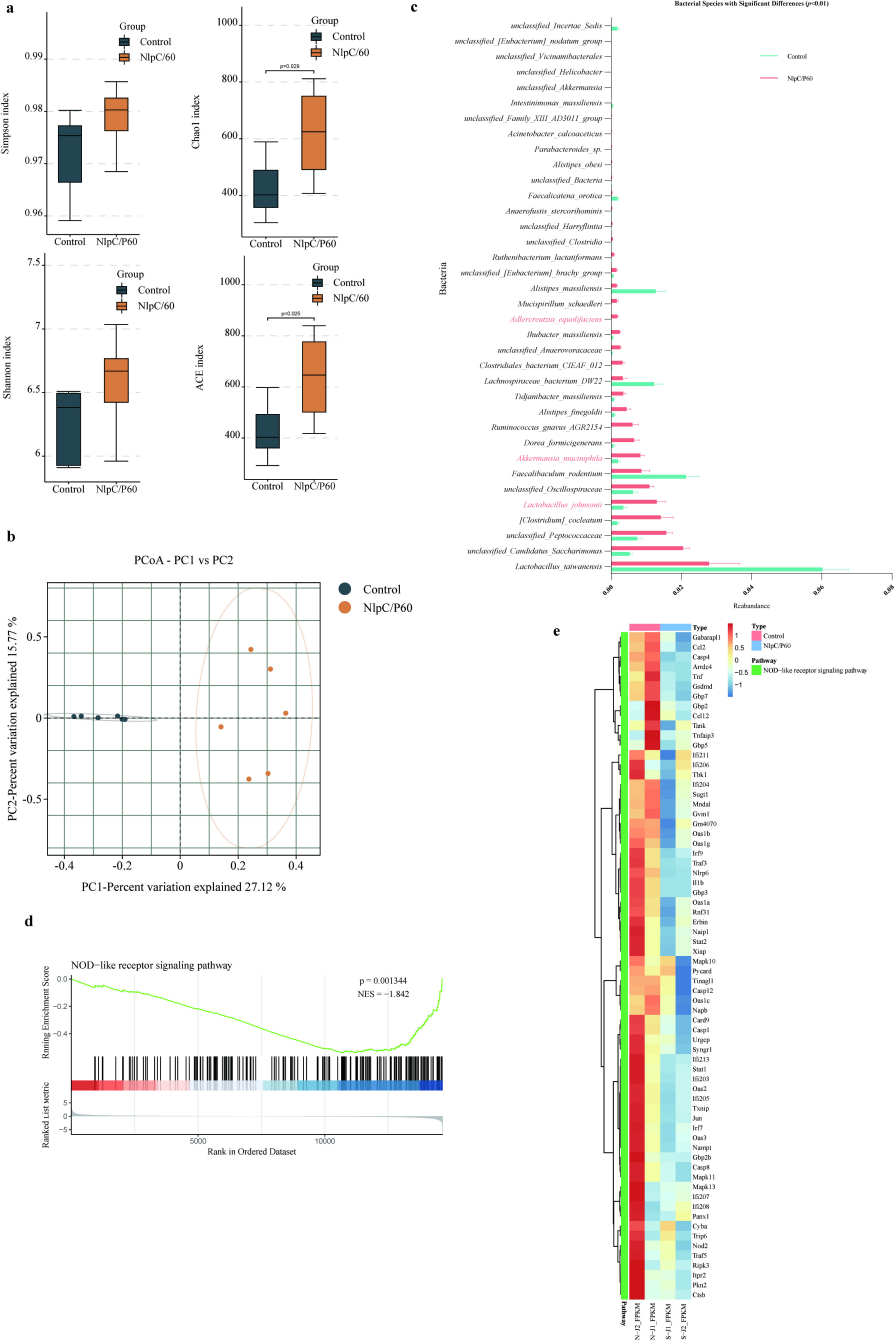
Effects of the NlpC/P60 family protein on gut microbiota and colon. **(a)** Differential Alpha Diversity Analysis (Each group: *n* = 6, statistical significance assessed using Student's *t*-test). **(b)** The principal coordinate analysis (PCoA) was conducted using the binary jaccard distance metric. **(c)** Metastats analysis was employed. The figure displays the results with significant differences at the species level between the groups (*p* < 0.01). **(d)** GSEA analysis of colon tissue transcriptomes between control and treatment groups. **(e)** Gene expression heatmap of the NOD-like receptor signaling pathway in colon tissues between control and NlpC/P60 groups.

Label-free quantitative proteomic analysis of *E. faecium* fermentation broth revealed that the NlpC/P60 family protein was the most abundant protein (Supplementary Figure S4). Literature reports suggest that its homologous proteins can activate innate immunity, promote an immune therapy-favorable microenvironment, and enhance immunotherapy efficacy (Griffin et al., 2021). They may also strengthen host immunity and suppress bacterial pathogens (Rangan et al., 2016; Kim et al., 2019). Therefore, we further investigated the potential role of the NlpC/P60 family protein in anti-aging. We conducted experiments using 18-month-old aged mice with an intervention period of 3 months, and the experimental design is illustrated in Figure 4a. As shown in Figure 4b, mice orally administered NlpC/P60 exhibited improved fur condition. The results of HE staining are shown in Figure 4c. The results indicated that the hepatic cords in the control group exhibited disordered structures, loose arrangement between hepatocytes, blurred boundaries, extensive fatty degeneration, pale and loose cytoplasm, round vacuoles, and partial nuclear pyknosis. In contrast, the NlpC/P60 group showed more uniform cytoplasm, reduced fatty degeneration, smaller round vacuoles, and clearer nuclei without significant pyknosis. Most renal tubules in the control group displayed dilated lumens and increased interstitial spacing, whereas the NlpC/P60 group exhibited narrower tubular lumens with regular morphology. Compared to the NlpC/P60 group, the spleen tissue in the control group showed structural disorganization, indistinct boundaries between white and red pulp, significantly reduced white pulp areas, and expanded red pulp regions with congestion and fibrosis. Additionally, tumor formations were observed in the liver and colon tissues of the control group (Supplementary Figure S7). IHC staining revealed reduced expression of the senescence marker p16^INK4A^ in both liver and kidney tissues in the NlpC/P60 group (Figure 4d). Western blot analysis further confirmed that oral administration of NlpC/P60 family protein decreased the expression levels of p53 and p16^INK4A^ in liver and kidney tissues (Figures 4e, f). These findings collectively demonstrate that oral NlpC/P60 family protein administration exerts anti-aging effects. The alpha diversity indices analysis revealed significant differences in Chao1 and ACE (Figure 5a), and the PCoA results of β-diversity are shown in the Figure 5b, demonstrating that the NlpC/P60 family protein could modulate the composition of gut microbiota. Metastats analysis of intergroup microbial composition differences demonstrated that the NlpC/P60 family protein significantly increased the abundance of *Akkermansia* (Depommier et al., 2019), *L. johnsonii* (Zhang et al., 2023) and *Adlercreutzia equolifaciens* (Oñate et al., 2023) which were regarded as common probiotics (Figure 5c). GSEA (Gene Set Enrichment Analysis) of colonic transcriptomes between the two groups revealed significant associations with the NOD-like receptor (Figure 5d). The heatmap displays the gene expression profiles of the NOD-like signaling pathway between the two groups (Figure 5e). The NlpC/P60 homologous protein could activate the NOD signaling pathway (Kim et al., 2019), and these experimental results are consistent with previous studies.

### Direct and indirect promotion of *A. muciniphila* by *E. faecium* components

**Figure 6:**
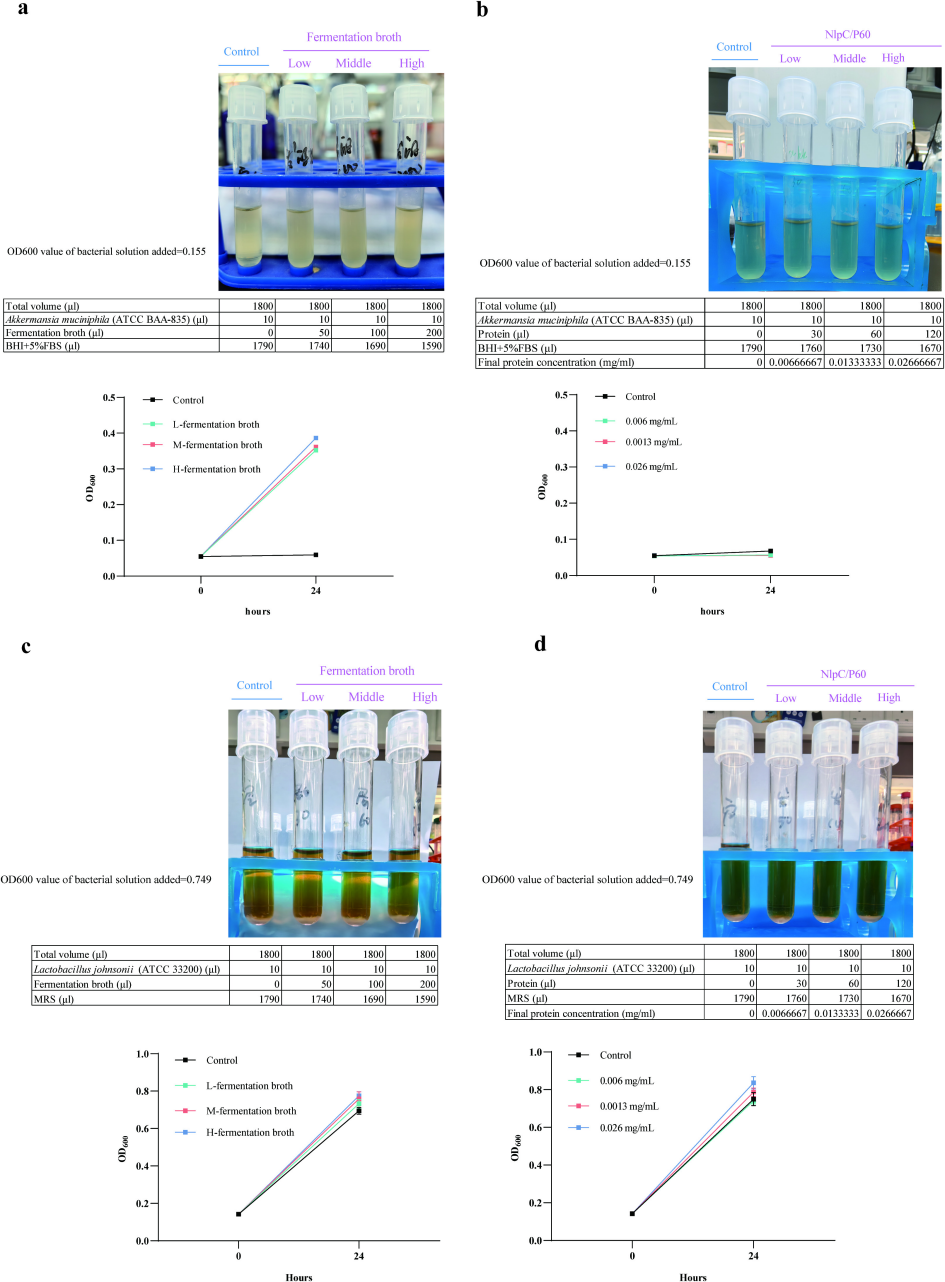
Effects of *E. faecium* fermentation broth and the NlpC/P60 family protein on *A. muciniphila* proliferation *in vitro*. **(a)** *E. faecium* fermentation broth significantly promoted *A. muciniphila* proliferation *in vitro*. **(b)** The NlpC/P60 family protein could not promote *A. muciniphila* proliferation *in vitro*. **(c)** *E. faecium* fermentation broth could not promote *L. johnsonii* proliferation *in vitro*. **(d)** The NlpC/P60 family protein could not promote *L. johnsonii* proliferation *in vitro*.

*In vitro* experiments demonstrated that *E. faecium* fermentation broth could directly promote the proliferation of *A. muciniphila*, whereas NlpC/P60 family protein showed no direct proliferative effect on this bacterium (Figures 6a, b). Both oral administration of *E. faecium* fermentation broth and NlpC/P60 family protein increased the abundance of *A. muciniphila* in the mouse gut. *In vitro* experiments confirmed that *E. faecium* fermentation broth directly promoted *A. muciniphila* proliferation, whereas NlpC/P60 family protein showed no such direct effect. These findings suggested that NlpC/P60 family protein likely promotes *A. muciniphila* growth indirectly by modulating the host's intestinal immune microenvironment rather than through direct microbial stimulation. We concurrently evaluated the growth-promoting effects of *E. faecium* fermentation broth and NlpC/P60 family protein on *L. johnsonii*. The results demonstrated that neither *E. faecium* fermentation broth nor NlpC/P60 family protein could directly promote the proliferation of *L. johnsonii* (Figures 6c, d).

## Discussion

This study demonstrates that *E. faecium* fermentation broth exhibits anti-aging effects in multiple organs of aged mice and significantly increases the abundance of *Akkermansia*. *A. muciniphila*, a “star probiotic,” has been widely reported to ameliorate aging-related phenotypes by enhancing gut barrier function and modulating host metabolism and immune responses (Depommier et al., 2019; Bae et al., 2022; Cani et al., 2022). Our findings reveal that *E. faecium* fermentation broth effectively promotes the proliferation of *A. muciniphila*, suggesting its potential as an *A. muciniphila*-promoting agent. The use of inactivated probiotics or their metabolites naturally avoids risks associated with live bacteria, such as mutations during growth and replication, as well as antibiotic resistance gene transfer (Doron and Snydman, 2015; Wang et al., 2020). Thus, heat-killed probiotics and their fermentation derivatives offer enhanced safety for specific populations, including immunocompromised individuals and those with severe infections or gastrointestinal disorders. Additionally, our study shows that *E. faecium* fermentation broth increases the number of colonic goblet cells and colonic stem cells (Figures 3f, g), improving age-related functional constipation.

Metabolomic analysis in this study revealed that *E. faecium* fermentation broth is enriched with inositol and D-ribose. As a key insulin sensitizer, inositol has been demonstrated to ameliorate PCOS (polycystic ovary syndrome) and metabolic disorders (Celentano et al., 2016; Facchinetti et al., 2020). Meanwhile, D-ribose, a crucial precursor for ATP synthesis, exhibits potential benefits in improving cardiac function, restoring energy in cardiomyocytes, and aiding recovery in patients with heart disease, heart failure, and chronic fatigue (Omran et al., 2003; Seifert et al., 2017). These fermentation metabolites may exert synergistic anti-aging effects. Notably, our study also uncovered the novel potential of myo-inositol supplementation to promote hair regeneration. Furthermore, oral administration of myo-inositol significantly increased the abundance of beneficial probiotics such as *L. reuteri* and *L. johnsonii*.

The innate immune system plays a pivotal regulatory role in maintaining tissue homeostasis and promoting injury repair (Ming et al., 2025). Studies in *C. elegans* have demonstrated that innate immunity positively contributes to longevity by enhancing pathogen resistance (Garsin et al., 2003; Yunger et al., 2017). With advancing age, innate immune cells exhibit characteristic functional decline, manifested by reduced phagocytic activity, decreased ROS (reactive oxygen species) generation, and downregulated expression of PRRs (pathogen recognition receptors), leading to increased susceptibility to infections and diminished vaccine responsiveness (Dorshkind et al., 2009). Given the crucial role of the immune system in combating infections, suppressing tumorigenesis, and maintaining tissue homeostasis and repair, therapeutic interventions targeting age-related immune dysfunction are essential for healthy aging (Dorshkind et al., 2009). Notably, recent studies have shown that activation of mucosal TLR5 effectively extends lifespan and preserves health in both male and female mice (Lim et al., 2024).

The major innovation of this study lies in uncovering the dual regulatory effects of *E. faecium* fermentation broth components on *A. muciniphila*: (1) the fermentation broth directly promotes *A. muciniphila* proliferation both *in vitro* and *in vivo*, and (2) the secreted protein NlpC/P60 indirectly enhances intestinal *A. muciniphila* abundance, potentially through host immune modulation.

## Conclusion

*E. faecium fermentation* broth may contribute to lifespan extension and ameliorates multi-organ aging phenotypes in aged mice through its bioactive components, demonstrating superior efficacy over live bacterial interventions. The *E. faecium* fermentation broth contained various anti-aging components, such as inositol and D-ribose, and could directly promote the proliferation of the probiotic *A. muciniphila*. Its highly abundant secreted protein NlpC/P60 indirectly enhances the colonic abundance of probiotics such as *A. muciniphila* and *L. johnsonii*, possibly by activating the NOD-like receptor pathway to modulate host immunity and thereby reshape the gut microbiota. Furthermore, *E. faecium* fermentation broth alleviates senile constipation and improves intestinal function by increasing colonic goblet cell counts and stem cell numbers. The component myo-inositol within the broth promotes hair regeneration. Oral administration of the NlpC/P60 family protein effectively reduces expression of senescence markers (p53/p16^INK4A^) in hepatic and renal tissues and mitigates organopathological damage. Although our strain has shown a favorable safety profile in current animal studies, we deliberately chose to use cell-free fermentation broth to further minimize any potential risks—particularly in vulnerable or immunocompromised populations. The acellular supernatant eliminates concerns related to bacterial translocation, horizontal transfer of antibiotic resistance genes, and unintended gut colonization. While our findings demonstrate promising health benefits of the *E. faecium* fermentation broth in model organisms, translation to human use will require rigorous safety evaluation—particularly in older adults and immunocompromised individuals, who may be more susceptible to potential risks associated with bacterial-derived products. Future studies should prioritize comprehensive toxicological profiling, dose optimization, and controlled clinical trials to ensure both efficacy and safety in diverse populations.

## Limitation

In our lifespan analysis using aged mice, we employed a limited number of animals due to constraints in the availability of aged mice and adherence to animal welfare policies aimed at minimizing the use of experimental animals. We fully agree that a larger sample size would enhance the robustness and reliability of our conclusions. At the current stage of this study, we have not yet fully elucidated the specific active compounds responsible for promoting *A. muciniphila* growth. However, our mass spectrometry analyses of *E. faecium* fermentation broth had identified several potential bioactive components. Building upon these findings, we plan to expand the scope of *in vitro* and *in vivo* metabolite screening in subsequent studies to further characterize the active constituents and mechanistic basis of *E. faecium* fermentation broth. This study provides a promising foundation for developing clinical interventions, demonstrating that oral administration of *E. faecium* fermentation broth (rather than live bacteria) effectively and safely delays organismal aging while improving gut microbiota profiles.

## Data Availability

The DNA sequencing data date were saved to NCBI database, with the accession code PRJNA952984. The 16S sequencing data date were saved to NCBI database, with the accession code PRJNA1105361, PRJNA957479, PRJNA1248140 and PRJNA1230834. The RNA-seq data were saved to NCBI database, with the accession code PRJNA1105361, PRJNA957479 and PRJNA1230834.
