# Supplementary material for: Enterococcus faecium secreted the NlpC/P60 family protein to enhance host immunity and indirectly increases Akkermansia muciniphila for slowing aging
Source: Front Microbiol. 2026 Jan 29;17:1680593. doi: 10.3389/fmicb.2026.1680593 (PMC12894319; doi:10.3389/fmicb.2026.1680593)
Supplement: Supplementary file 1 [file Supplementary_file_1.docx]

**Supplementary Information**

**Materials**

| **Antibodies** | **Source** | **Identifier** | **Antibody working concentration** |
| --- | --- | --- | --- |
| LGR5 | Affinity | DF2816 | WB 1:1500 |
| IL10 | Affinity | DF6894 | WB 1:1500 |
| FOXP3 | Servicebio | GB112325 | WB 1:1000 |
| GAPDH | Affinity | AF7021-100 | WB 1:5000 |
| Goat Anti-Rabbit lgG(H+L) HRP | Affinity | S0001 | WB 1:3000, IHC 1:200 |
| Anti-CDKN2A/p16^INK4a^ | Abcam | ab189034 | WB 1:1000, IHC 1:500 |
| Anti-p53 antibody [POE316A] | Abcam | ab241566 | WB 1:1500 |

| **Chemicals** | **Source** | **Identifier** |
| --- | --- | --- |
| Diaminobenzidine (DAB) | Servicebio | G1212 |
| Citrate buffer pH 6.0 | Servicebio | G1202 |
| Anhydrous ethanol | Guangzhou Guanghua Sci-Tech Co., Ltd | 1.17113.023 |
| Phosphate-buffered saline | CORNING | 19117004 |
| Fetal bovine serum | Gibco | 1932595 |
| MRS culture medium | Huankai Microbial | 027315 |
| Brain Heart Infusion Broth | Huankai Microbial | 024053 |
| NGM culture medium | SHANGDONG TUOPO BIOL-ENGINEERING CO., LTD. | M2328 |
| CaCl_2_ | Shanghai Aladdin Biochemical Technology Co., Ltd. | C399250 |
| MgSO_4_ | Shanghai Aladdin Biochemical Technology Co., Ltd. | M485462 |
| Sodium sulfide nonahydrate (Na_2_S·9H_2_O) | Shanghai Aladdin Biochemical Technology Co., Ltd. | S101399 |
| Myo-Inositol | Shanghai Aladdin Biochemical Technology Co., Ltd. | I656931 |
| Protease And Phosphatase Inhibitor Cocktail For Mammalian Cell And Tissue Extracts | Solarbio | P1261 |
| BCA Protein Assay Kit | Solarbio | PC0020 |

| **Stain** | **Source** | **Identifier** |
| --- | --- | --- |
| *Akkermansia Muciniphila* | ATCC | ATCC BAA-835 |
| *Lactobacillus johnsonii* | biobw | Bio-03600/ATCC 33200 |
| *Caenorhabditis elegans* | SunyBiotech | - |
| OP50 | SunyBiotech | - |

**Deposited Data**

| **Data type** | **Detail** | **The accession code of NCBI database** |
| --- | --- | --- |
| DNA sequencing date | Complete map of the whole genome of *E. faecium* | PRJNA952984 |
| 16S sequencing data | Bacterial microbiome of colonic contents of aged mice fed with *E. faecium* fermentation broth. | PRJNA1105361 |
| RNA sequencing data | Transcriptomic data of the spleen in aged mice fed with *E. faecium* fermentation broth. | PRJNA1105361 |
| 16S sequencing data | Bacterial microbiome of colonic contents of KM aged constipated mice fed with *E. faecium* fermentation broth | PRJNA957479 |
| RNA sequencing data | Transcriptomic data of the colon in KM aged constipated mice administered with *E. faecium* fermentation broth via gavage. | PRJNA957479 |
| 16S sequencing data | Bacterial microbiome of colonic contents of KM aged mice fed with inositol | PRJNA1248140 |
| 16S sequencing data | Bacterial microbiome of colonic contents of aged mice fed with NlpC/P60 family protein. | PRJNA1230834 |
| RNA sequencing data | Transcriptomic data of the colon in aged mice fed with NlpC/P60 family protein. | PRJNA1230834 |

**Supplementary Methods**

**Prediction of protein-coding genes in *E. faecium***

Gene prediction for the entire gene sequence was conducted using GeneMarkS software (John B et al., 2001). GeneMarkS is utilized for protein-coding gene prediction in bacterial genomes. The prediction method of this software involves establishing corresponding statistical models through GeneMark.hmm. It utilizes frequency matrices of nucleotide usage within the sequence as a foundation to predict potential coding regions within the sequence. This approach enhances the recognition of gene translation sites and reduces the false positive rate in gene prediction. Additionally, this software is recognized by NCBI as a prokaryotic coding gene prediction tool. The amino acid sequences of all open reading frames (ORFs) are shown in supplementary table.

**Lifespan experiment of *Caenorhabditis elegans***

The experiment is divided into two groups: a control group (OP50) and an *E. faecium* group (OP50+ *E. faecium*). *E. faecium* was anaerobic cultured in MRS broth at 37 °C for 24 h and continuously cultured to four generations as test bacterial solution. *E. coli* OP50 was incubated in Luria Bertani (LB) broth at 37°C for 12 h with a shaking at 120 rpm, with OD_600_ being approximately equal to 0.6. For preparing bacterial plates for the worms feeding, *E. faecium* was collected through centrifugation at 4000 × g for 10 min, and washed with M9 buffer (3g KH_2_PO_4_, 6g Na_2_HPO_4_, 5g NaCl and 1mL 1M MgSO_4_) twice, and then resuspended in *E. coli* OP50 until its OD_600_≈1 to a final concentration which were used as the bacteria supply. 60 μL of the bacteria solution was inoculated on 60 mm NGM plate. Forty L4-stage N2 nematodes are transferred to petri dishes containing bacteria for each group. To ensure that the medication remains effective throughout the experiment, the nematodes are transferred to new petri dishes daily and gently stimulated with a platinum wire. The number of surviving nematodes is recorded daily until the last nematode dies.

**Transformation of plasmid and small-scale expression**

Add 0.5 μl of plasmid into 100 μl of BL21(DE3) competent cells and place on ice for 20 minutes. Perform heat shock at 42°C for 90 seconds, then quickly place on ice for 2 minutes. Add 500 μl of LB broth. Incubate with shaking at 37°C and 220 rpm for 30 minutes. Spread 150 μl of bacterial suspension onto an LB plate containing 100 μg/ml Ampicillin. Invert and incubate at 37°C overnight. The next morning, pick three single colonies from the plate and inoculate them into test tubes containing 1.5 ml of LB broth with 100 μg/ml Ampicillin. Incubate with shaking at 37°C and 220 rpm until the OD_600_ reaches approximately 0.6. Remove two 500 μl aliquots of bacterial suspension and add IPTG to a final concentration of 0.1 mM. Induce at 220 rpm for 3 hours at 22°C and 37°C, respectively. Centrifuge at 10,000 rpm for 2 minutes to remove the supernatant and collect the bacterial pellets. Resuspend the pellets in 50 μl of TBS, add 10 μl of *E.coli* Lysis Buffer to lyse the cells. Centrifuge and collect the whole bacterial lysate and supernatant for electrophoresis to detect protein expression.

**Protein purification**

Inoculate glycerol bacteria at a ratio of 1:1000 into 50 ml of LB broth containing 100 μg/ml Ampicillin, and incubate with shaking at 37°C and 220 rpm overnight. Inoculate the overnight-cultured bacteria at a ratio of 1:100 into 500 ml of LB broth containing 100 μg/ml Ampicillin, and incubate with shaking at 37°C and 220 rpm until the OD_600_ reaches 0.6-0.8. Prepare two bottles with a total volume of 1000 ml. Add the inducer IPTG to a final concentration of 0.1 mM in the 500 ml culture, and incubate at 37°C and 220 rpm for 4 hours. Centrifuge at 5000 rpm for 5 minutes at 4°C to remove the supernatant and collect the fermented bacterial pellets. Store at -20°C. Bacterial lysis: Resuspend the bacterial pellets collected from 500 ml of culture in 25 mM Tris, 500 mM NaCl, pH 7.4, and add 3 ml of bacterial lysis buffer for lysis. Purification of soluble proteins using Smart-NI (2 ml)：Centrifuge the lysed bacteria at 12000 rpm for 10 minutes at 4°C, and collect the supernatant for nickel column purification using Smart-NI as the filler. Equilibration buffer: 25 mM Tris, 500 mM NaCl, pH 7.4. (Equilibration buffer: 25 mM Tris, 500 mM NaCl, pH 7.4. Endotoxin removal solution: 25 mM Tris, 500 mM NaCl, pH 7.4, 1% TritonX-100.) Endotoxin removal: After loading the sample onto the filler during the purification process, wash with 20 column volumes of endotoxin removal solution to remove endotoxins. Then wash with 10 column volumes of equilibration buffer, and finally elute with a gradient of elution buffer. (Elution buffer: 25 mM Tris, 500 mM NaCl, 250 mM imidazole, pH 7.4. Elute with a gradient of 2 mM/20 mM/50 mM/250 mM imidazole.)

**Protein sequence of** **NlpC/P60 family protein**

VKKSLISAVMVCSMTLTAVASPIAAAADDFDSQIQQQDQKIADLKNQQADAQSQIDALESQVSEINTQAQDLLAKQDTLRQESAQLVKDIADLQERIEKREDTIQKQAREAQVSNTSSNYIDAVLNADSLADAIGRVQAMTTMVKANNDLMEQQKQDKKAVEDKKAENDAKLKELAENQAALESQKGDLLSKQADLNVLKTSLAAEQATAEDKKADLNRQKAEAEAEQARIREQQRLAEQARQQAAQEKAEKEAREQAEAEAQATQASSAAQSSATEESSAAQSSTTEESSSAAQSSTTEESTTAPESSTTEESTTAPESSTIEESTTAPESSTTEESTTVPESSTTEESTTVPESSTEESTTPAPTTPSTDQSVDPGNGTGSNATNNTTNTTPTPTPTPTPSGSVNGAAIVAEAYKYIGTPYVWGGKDPSGFDCSGFTRYVYLQVTGRDIGGWTVPQESAGTKISVSQAKAGDLLFWGSPGGTYHVAIALGGGQYIHAPQPGESVKVGSVQWFAPDFAVSM

**Labeled protein sequence of NlpC/P60 family protein（His tag and Strep tag）**

MHHHHHHADDFDSQIQQQDQKIADLKNQQADAQSQIDALESQVSEINTQAQDLLAKQDTLRQESAQLVKDIADLQERIEKREDTIQKQAREAQVSNTSSNYIDAVLNADSLADAIGRVQAMTTMVKANNDLMEQQKQDKKAVEDKKAENDAKLKELAENQAALESQKGDLLSKQADLNVLKTSLAAEQATAEDKKADLNRQKAEAEAEQARIREQQRLAEQARQQAAQEKAEKEAREQAEAEAQATQASSAAQSSATEESSAAQSSTTEESSSAAQSSTTEESTTAPESSTTEESTTAPESSTIEESTTAPESSTTEESTTVPESSTTEESTTVPESSTEESTTPAPTTPSTDQSVDPGNGTGSNATNNTTNTTPTPTPTPTPSGSVNGAAIVAEAYKYIGTPYVWGGKDPSGFDCSGFTRYVYLQVTGRDIGGWTVPQESAGTKISVSQAKAGDLLFWGSPGGTYHVAIALGGGQYIHAPQPGESVKVGSVQWFAPDFAVSMWSHPQFEK

**
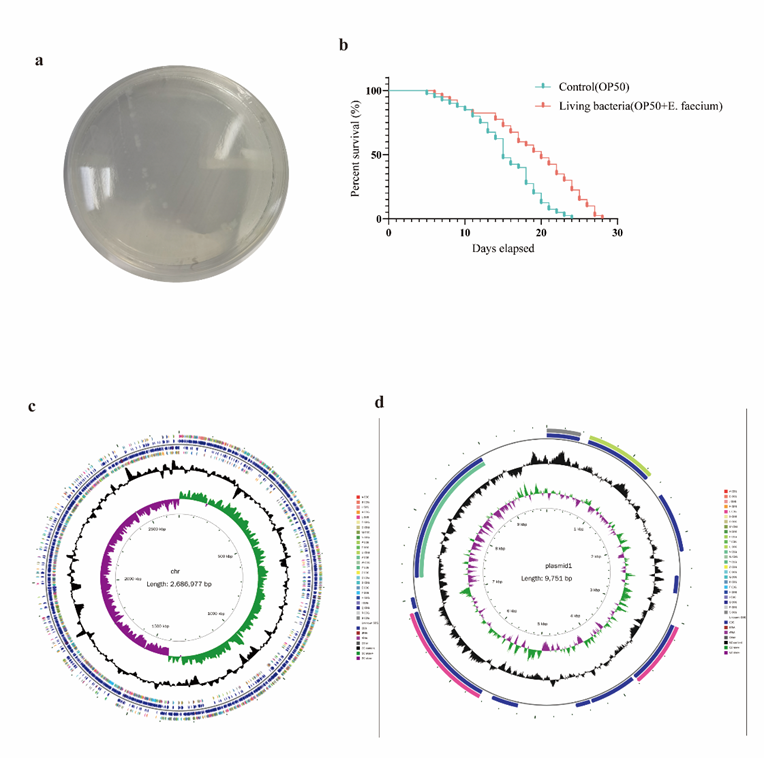
Supplementary Figure 1. Information about *E. faecium***

(a)The bacterial colony was moist, milky and translucent, milky white on MRS Medium, and liquid broth culture had an aromatic odor, which could significantly prolong the life of nematode. (b) survival curves of *C. elegans* fed with living *E. faecium*. (c) Genomic cycle Map (chr) (d) Genomic cycle Map (plasmid1). The genome sequencing data obtained in this study were saved to NCBI database, with the accession code PRJNA952984. Firstly, the genomic sequence, gene predictions, and predictions of non-coding RNAs are integrated into a standard GenBank (GBK) format file. Then, the genomic circle plot is drawn using cgview (Stothard P and Wishart DS, 2005). Finally, the plot is edited using Photoshop CS. From the inside out, the first circle represents the scale; the second circle represents GC Skew; the third circle represents GC content; the fourth and seventh circles represent the COG categories for each CDS; and the fifth and sixth circles represent the positions of CDS, tRNA, and rRNA on the genome.


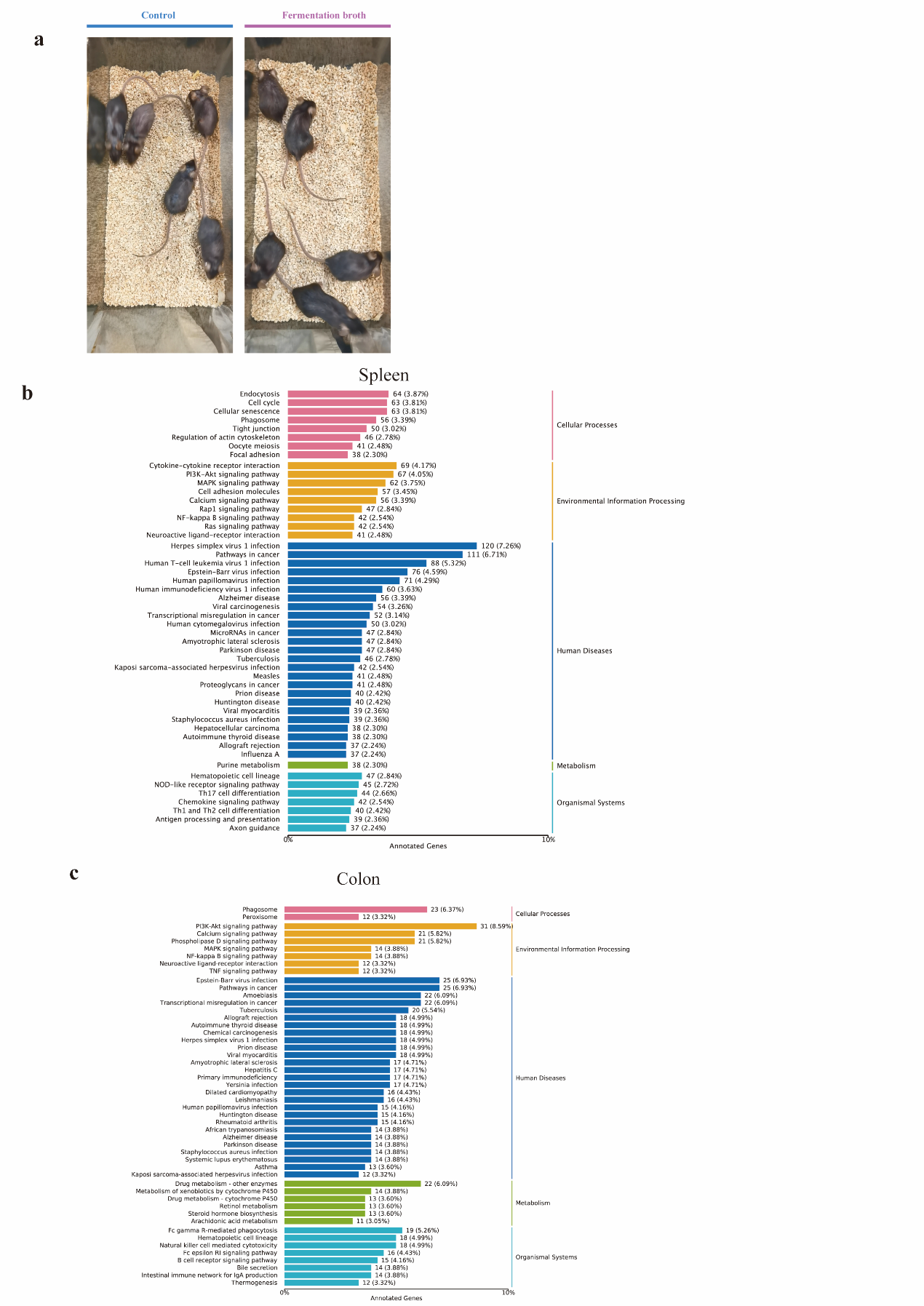


**Supplementary Figure 2 The phenotypic data of aged mice after oral administration of *E. faecium* fermentation broth**

(a) The changes in hair condition of mice after oral administration of *E. faecium* fermentation broth. (b) KEGG enrichment analysis results of splenic transcriptome in aged mice following oral administration of *E. faecium* fermentation broth. (c) KEGG enrichment analysis results of colonic transcriptome in KM aged constipated mice following oral administration of *E. faecium* fermentation broth.


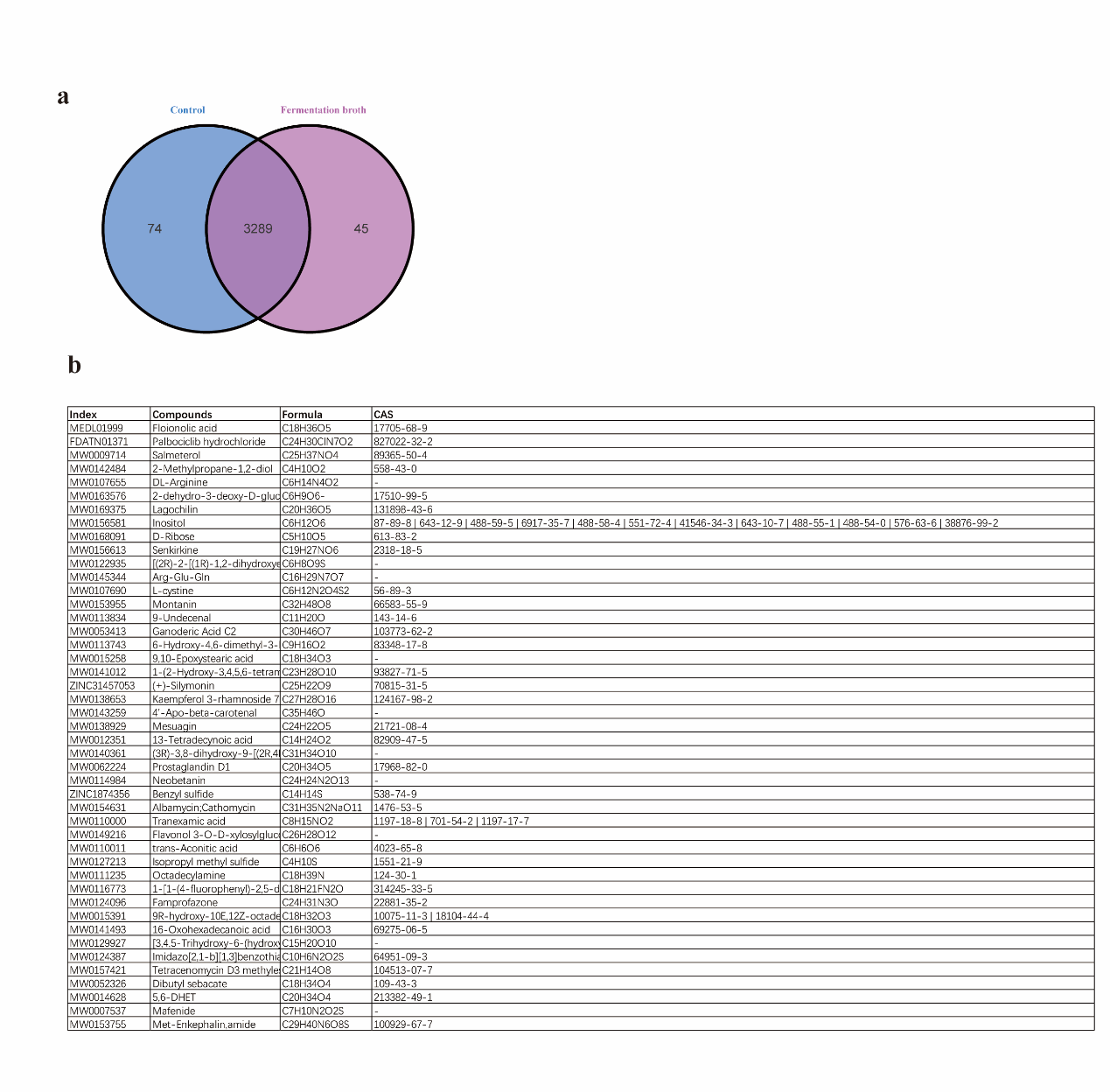


**Supplementary Figure 3 *E. faecium* fermentation broth differential metabolite (identified by non-targeted metabolomics)**

(a) Venn diagram of identification results for MRS medium and *E. faecium* fermentation broth (identified by non-targeted metabolomics). (b) There are 45 newly produced metabolites after fermentation (identified by non-targeted metabolomics).


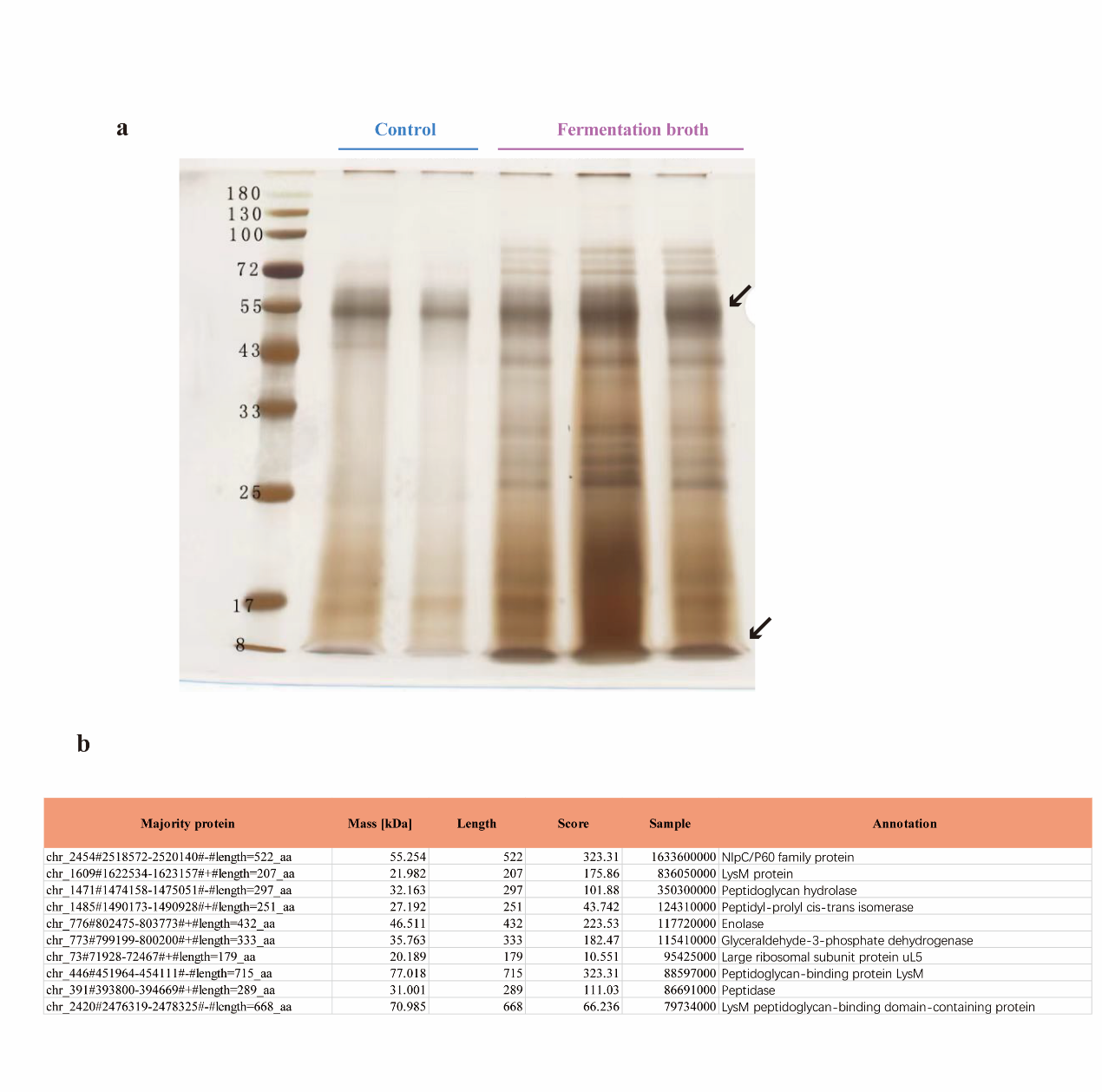


**Supplementary Figure 4 *E. faecium* fermentation broth differential metabolite (identified by label-free quantitative proteomic)**

(a) Silver staining was performed on MRS medium and *E. faecium* fermentation broth to observe the molecular weight distribution of newly produced proteins or peptides. (b) Label-free quantitative proteomic results (top 10 identified proteins by abundance).


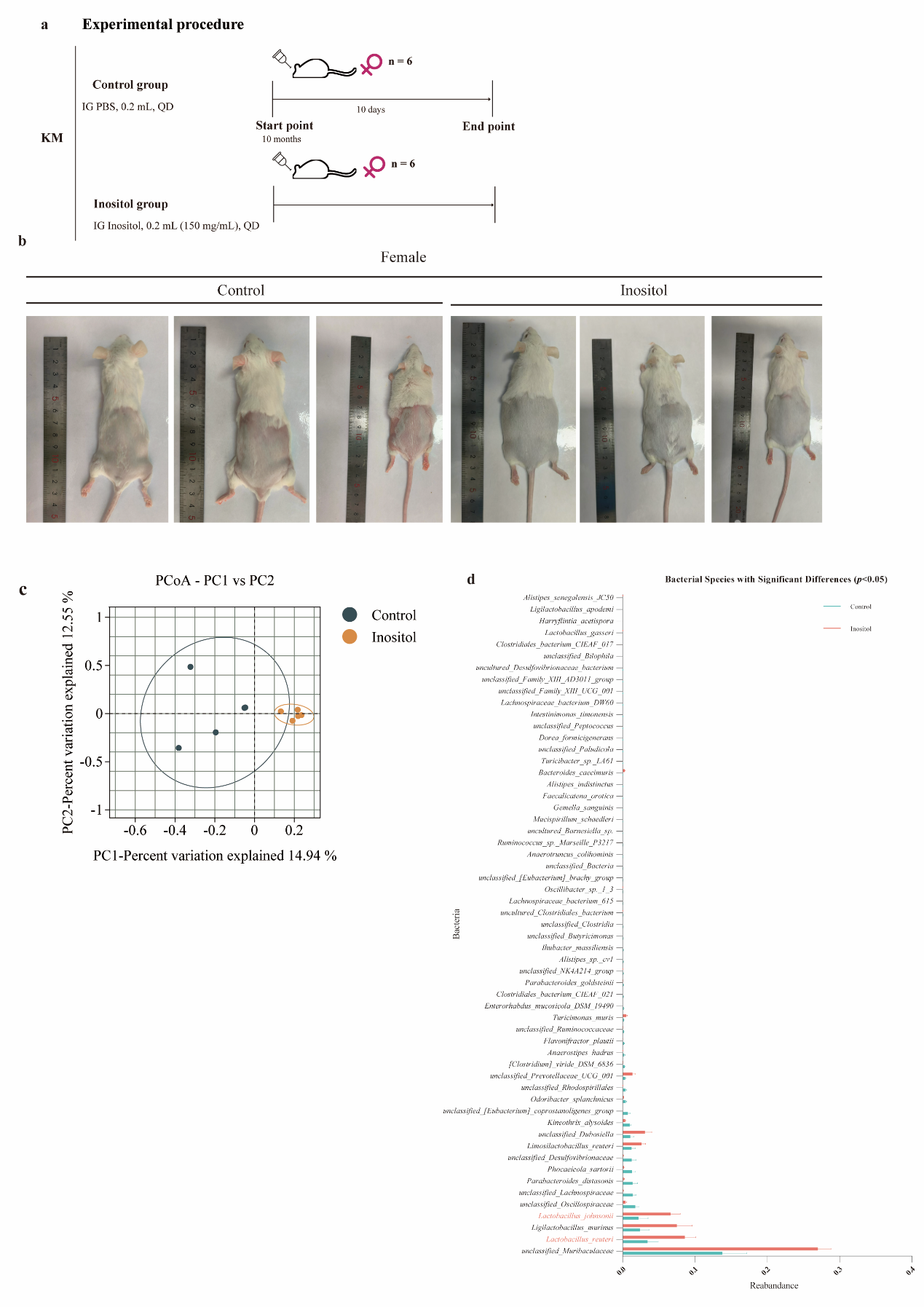


**Supplementary Figure 5 Inositol, a metabolite derived from *E. faecium* fermentation broth, promotes hair growth.**

(a) Schematic of the animal experimental design and group allocation. (b) Hair growth status in aged mice. (c) The principal coordinate analysis (PCoA) was conducted using the binary jaccard distance metric. (d) Metastats analysis was employed. The figure displays the results with significant differences at the species level between the groups (*p* < 0.05).


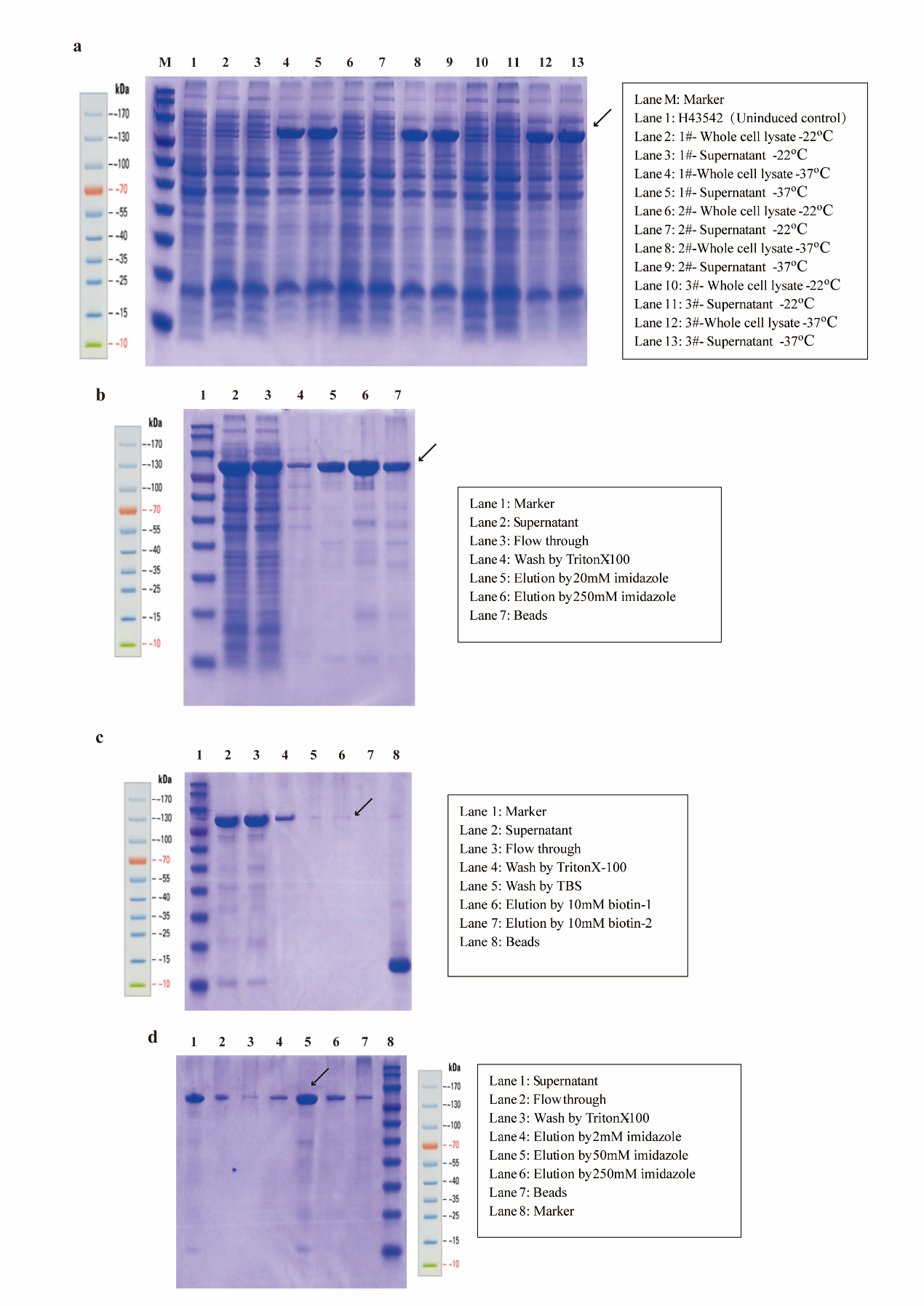


**Supplementary Figure 6 Protein purification of NlpC/P60 family protein**

(a) All three monoclonal cultures of H43542, after induction, exhibited the target protein with an apparently larger molecular weight. The protein expression level was high, and it was soluble. (b) The target protein exhibits high expression levels and is soluble. It can bind to a nickel column, and the eluted protein shows no significant degradation, with a relatively high purity. However, there are a few impurity bands present. (c) The protein eluted from the nickel column was further purified using STarm Streptactin Beads 4FF as the filler. The equilibration buffer consisted of 25 mM Tris, 500 mM NaCl, and pH 7.4, while the elution buffer contained 25 mM Tris, 500 mM NaCl, pH 7.4, and 10mM biotin. As shown in the figure, the target protein barely bound to the STarm filler. (d) The target protein underwent another purification process using a nickel column, with an imidazole gradient wash. The final eluted protein exhibited a high purity of approximately 90%. The protein eluted with 50 mM imidazole was then dialyzed against a solution containing 20 mM PB, 150 mM NaCl, at a pH of 7.4. The concentration of the target protein was 0.4 mg/ml, and it was stored in a buffer consisting of 20 mM PB, 150 mM NaCl, 5% glycerol, at a pH of 7.4.


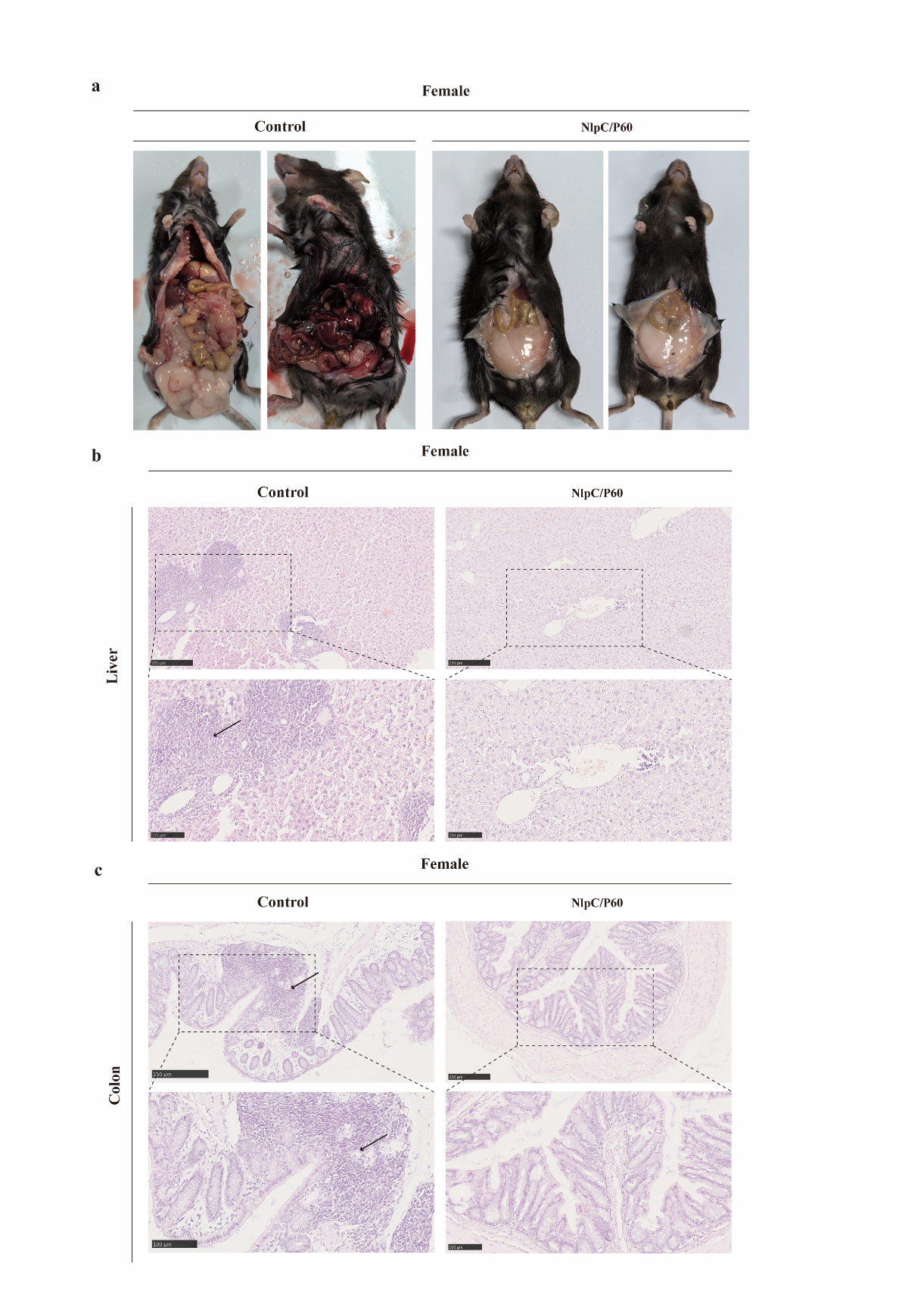


**Supplementary Figure 7 The phenotypes and pathological sections of aged mice after oral administration of NlpC/P60 family protein.**

(a) Abdominal dissection findings in aged mice following oral administration of NlpC/P60 family protein. (b) HE staining results of liver sections in aged mice following oral administration of NlpC/P60 family protein. (c) HE staining results of colon sections in aged mice after oral administration of NlpC/P60 family protein.
